# Monitoring and Management of Home-Quarantined Patients With COVID-19 Using a WeChat-Based Telemedicine System: Retrospective Cohort Study

**DOI:** 10.2196/19514

**Published:** 2020-07-02

**Authors:** Hui Xu, Sufang Huang, Chun Qiu, Shangkun Liu, Juan Deng, Bo Jiao, Xi Tan, Ling Ai, Yaru Xiao, Mirko Belliato, Li Yan

**Affiliations:** 1 Department of Anesthesiology Tongji Hospital of Tongji Medical College Huazhong University of Science and Technology Wuhan China; 2 Department of Emergency Medicine Tongji Hospital of Tongji Medical College Huazhong University of Science and Technology Wuhan China; 3 Lazaridis School of Business and Economics Wilfrid Laurier University Waterloo, ON Canada; 4 Anestesia e Rianimazione 1 Foundation IRCCS Policlinico San Matteo Pavia Italy

**Keywords:** telemedicine system, home quarantine, quarantine management assessment, progressive COVID-19 patients, COVID-19

## Abstract

**Background:**

Most patients with coronavirus disease (COVID-19) who show mild symptoms are sent home by physicians to recover. However, the condition of some of these patients becomes severe or critical as the disease progresses.

**Objective:**

The aim of this study was to evaluate a telemedicine model that was developed to address the challenges of treating patients with progressive COVID-19 who are home-quarantined and shortages in the medical workforce.

**Methods:**

A telemedicine system was developed to continuously monitor the progression of home-quarantined patients with COVID-19. The system was built based on a popular social media smartphone app called WeChat; the app was used to establish two-way communication between a multidisciplinary team consisting of 7 medical workers and 188 home-quarantined individuals (including 74 confirmed patients with COVID-19). The system helped patients self-assess their conditions and update the multidisciplinary team through a telemedicine form stored on a cloud service, based on which the multidisciplinary team made treatment decisions. We evaluated this telemedicine system via a single-center retrospective study conducted at Tongji Hospital in Wuhan, China, in January 2020.

**Results:**

Among 188 individuals using the telemedicine system, 114 (60.6%) were not infected with COVID-19 and were dismissed. Of the 74 confirmed patients with COVID-19, 26 (35%) recovered during the study period and voluntarily stopped using the system. The remaining 48/76 confirmed patients with COVID-19 (63%) used the system until the end of the study, including 6 patients whose conditions progressed to severe or critical. These 6 patients were admitted to hospital and were stabilized (one received extracorporeal membrane oxygenation support for 17 days). All 74 patients with COVID-19 eventually recovered. Through a comparison of the monitored symptoms between hospitalized and nonhospitalized patients, we found prolonged persistence and deterioration of fever, dyspnea, lack of strength, and muscle soreness to be diagnostic of need for hospitalization.

**Conclusions:**

By continuously monitoring the changes in several key symptoms, the telemedicine system reduces the risks of delayed hospitalization due to disease progression for patients with COVID-19 quarantined at home. The system uses a set of scales for quarantine management assessment that enables patients to self-assess their conditions. The results are useful for medical staff to identify disease progression and, hence, make appropriate and timely treatment decisions. The system requires few staff to manage a large cohort of patients. In addition, the system can solicit help from recovered but self-quarantined medical workers to alleviate shortages in the medical workforce and free healthy medical workers to fight COVID-19 on the front line. Thus, it optimizes the usage of local medical resources and prevents cross-infections among medical workers and patients.

## Introduction

Since its outbreak in December of 2019, coronavirus disease (COVID-19) has spread worldwide, causing more than 5 million infections and tens of thousands of deaths in the course of three months [1]. In cities that were severely affected by COVID-19, such as Wuhan (China), Lombardy (Italy), and New York City (United States) [1], local medical capacities were quickly depleted by large numbers of patients who hurried to hospitals for treatment. Many medical workers were infected, and medical care supplies were further exhausted [2]. To prevent collapse of the global health care system, many countries have advocated for infected patients with mild symptoms to stay home and self-quarantine [3]. However, it has been observed that the condition of some home-quarantined patients becomes severe or critical as the disease progresses. Home quarantine can delay timely treatment and hospitalization of these patients, which may lead to their death.

In this paper, we report a telemedicine model that we developed to address the challenges outlined above. This telemedicine system enabled close monitoring of 74 home-quarantined COVID-19 patients from January 6 to 31, 2020. Of the 74 patients, 6 (8%) were admitted to hospital when signs of deterioration were detected by the system. One patient received extracorporeal membrane oxygenation (ECMO) treatment for 17 days. All 74 patients recovered.

The telemedicine model was built based on WeChat, a popular smartphone app for instant messaging and social media. The WeChat app established two-way communication between the home-quarantined patients and a multidisciplinary team. The multidisciplinary team contacted the patients regularly to receive information updates. The information was subsequently analyzed to determine the latest status of the patients. Home-quarantined patients could also initiate communication to report any abnormalities in addition to receiving feedback about their medical conditions. The system we developed is the first to quantify subjective symptom descriptions with objective numerical scales. In addition, this telemedicine system helps alleviate the workload of overwhelmed medical staff [4], as we found that excessive laboratory data and physical examinations were not strictly necessary to identify the prognoses of patients with mild symptoms. Furthermore, the telemedicine model minimizes the risk of infection among caregiving staff by reducing their direct physical contact with patients. In this paper, we summarize the procedures of the telemedicine model and present clinical evidence of its success.

## Methods

We evaluated the telemedicine system via a single-center retrospective study conducted at Tongji Hospital in Wuhan, China, between January 6 and January 31, 2020. The study was approved by the Tongji Hospital Ethics Committee before data were collected retrospectively.

### Procedure and Information Flow of the Telemedicine System

**Figure 1:**
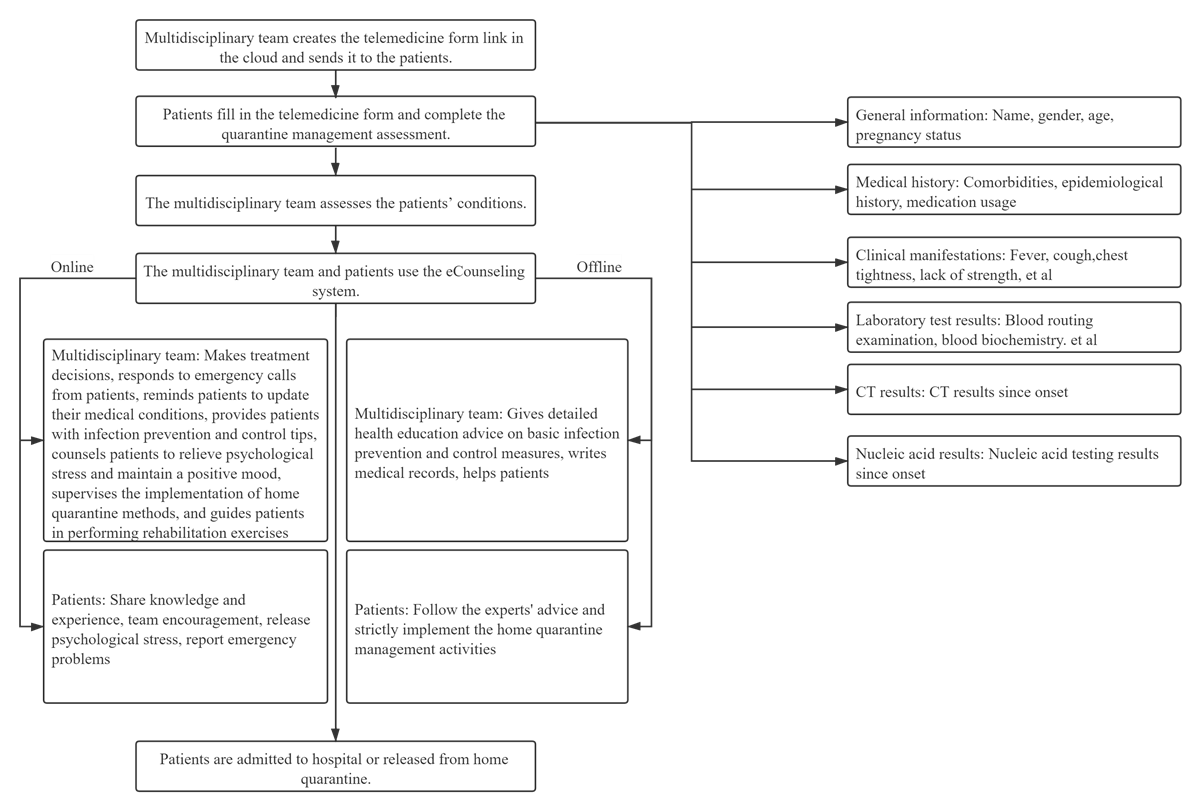
Flowchart of the telemedicine system, including the steps to enroll a patient, conduct self-assessment via the quarantine management assessment, and update conditions using the eCounseling system. CRP: C-reactive protein; CT: computed tomography; eCounseling: electronic counseling; PCT: procalcitonin.

Below, we outline the procedure of the telemedicine system. When a patient signed up for the telemedicine system, the multidisciplinary team created a patient-specific telemedicine form in the cloud. The telemedicine form was then sent to the patient through a link via the WeChat app to the patient’s mobile phone or by email. The patient began the process by filling in the telemedicine form (Figure 1).

The patient first answered a set of standardized questions, which included general demographic information (name, gender, age, occupation, and pregnancy status), medical history (comorbidities, epidemiological history within the past 14 days, and medication usage), clinical manifestations (eg, fever, cough, chest tightness, lack of strength), various laboratory tests, computed tomography (CT) images, and nucleic acid test results for severe acute respiratory syndrome coronavirus 2 (SARS-CoV-2) detection (Figure 1).

Next, the patient underwent quarantine management assessment. This assessment was based on a set of medical observation scales developed by the multidisciplinary team based on both a literature review [5-9] and input from 34 medical experts from our medical school or its affiliated hospitals (Multimedia Appendix 1). The quarantine management assessment helped the multidisciplinary team assess the patients’ conditions.

Based on the assessment, the multidisciplinary team determined whether the patient should be hospitalized immediately or placed under medical observation at home. If the patient was placed under medical observation, the patient would then begin using the electronic counseling (eCounseling) system, which facilitated close observation by and efficient communication with the multidisciplinary team. Specifically, the patient was required to update their conditions on a daily basis using the telemedicine form. Due to the convenience provided by the cloud service, the information could be instantly accessed by pertinent multidisciplinary team members. They subsequently provided feedback and guidance on the telemedicine form, which then could also be accessed by the patient immediately.

In addition, the patient was invited to join a WeChat group consisting of patients participating in the telemedicine system and multidisciplinary team members. In this way, the patient could receive health tips from the multidisciplinary team and conduct a group chat with other patients and multidisciplinary team members. The patient could also initiate a one-to-one chat or telephone call with any multidisciplinary team member using built-in functions in the WeChat app.

**Figure 2:**
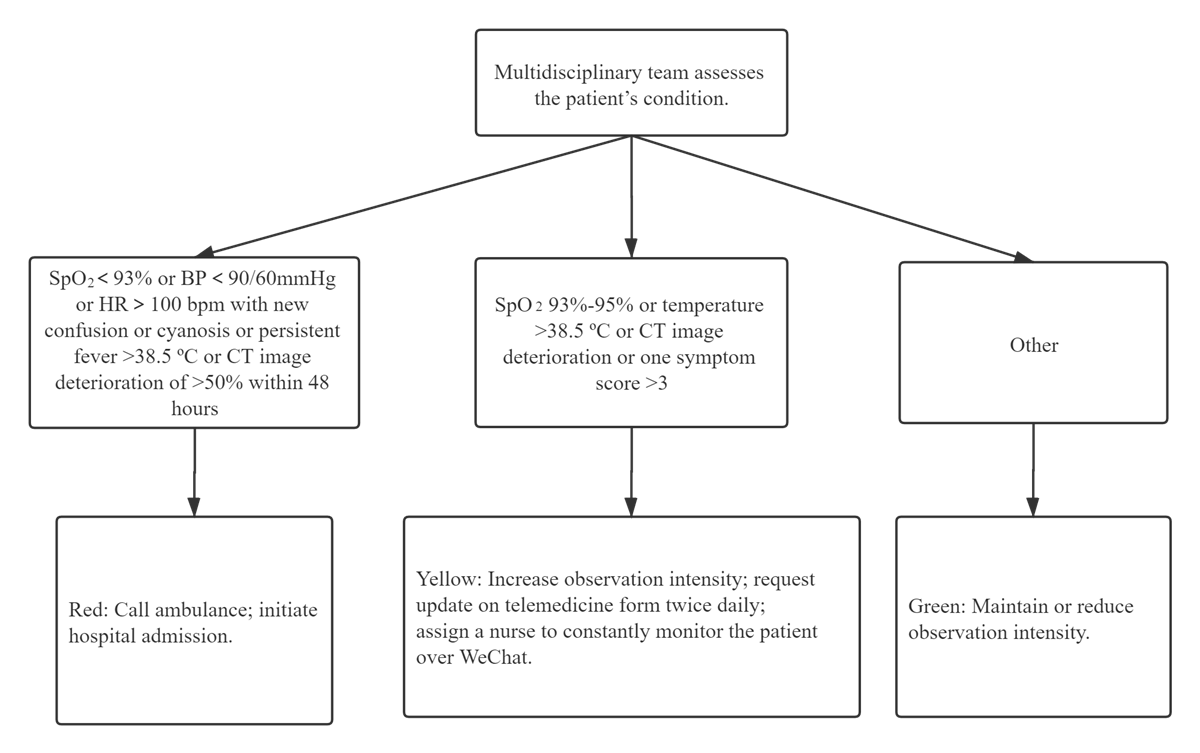
Decision tree of patient treatment for MDT based on patients’ condition updates via the eCounseling system. ºC: degrees Celsius; BP: blood pressure; bpm: beats per minute; CT: computed tomography; mmHg: millimeters of mercury; SpO2: oxygen saturation.

**Figure 3:**
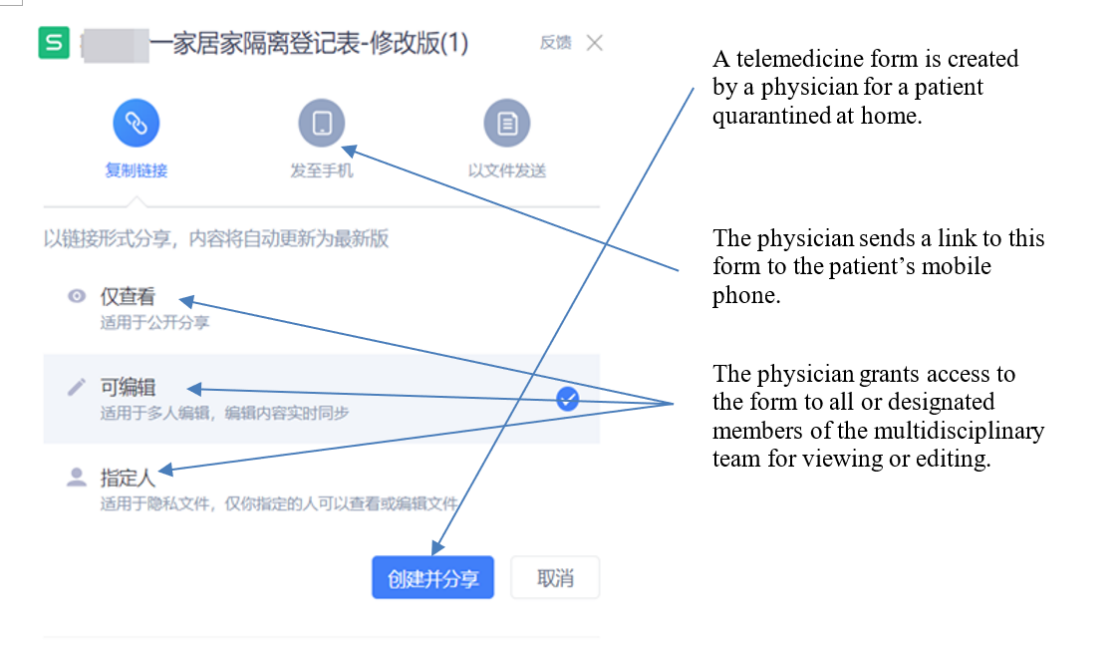
Screenshot of the creation of the telemedicine form by the multidisciplinary team (in Chinese, with English annotations).

**Figure 4:**
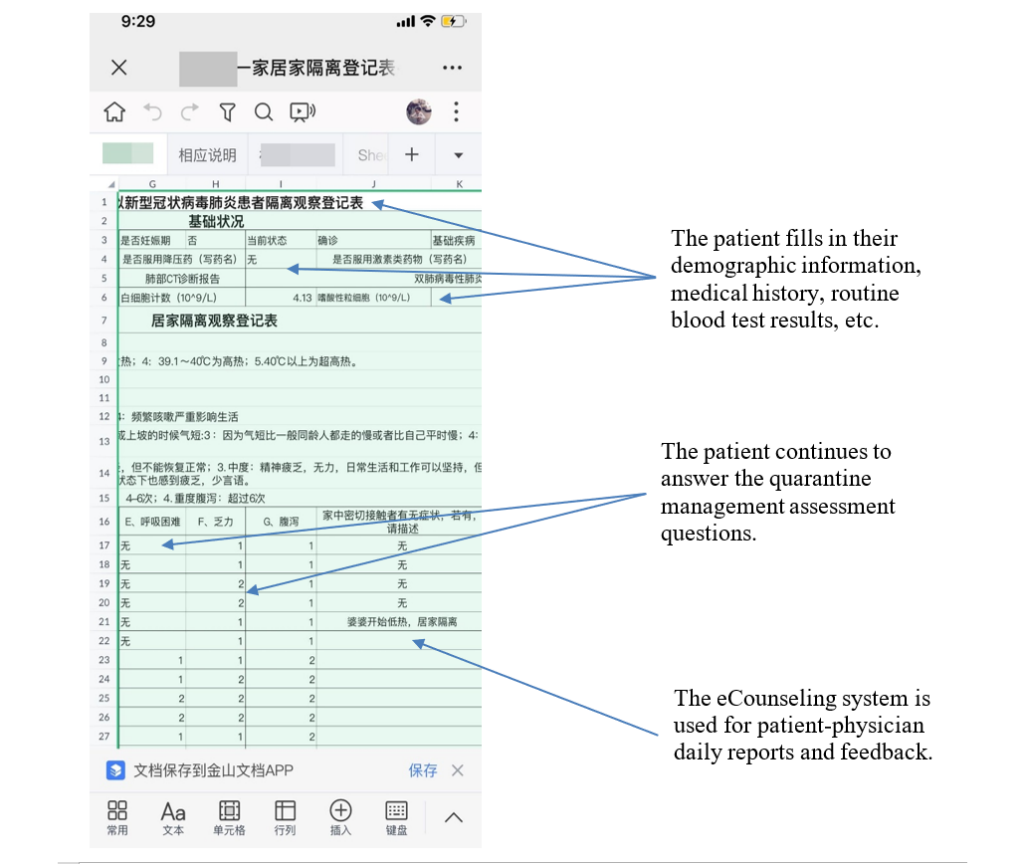
Screenshot of the patient interface of the telemedicine form (in Chinese, with English annotations). eCounseling: electronic counseling.

The multidisciplinary team adjusted their observational attention based on the progression of each patient’s condition. If a patient’s condition continued to worsen (eg, repeated and persistent fever >38.5 degrees Celsius, cyanosis or CT image deterioration >50% within 48 hours) or their mental state scores continued to decline, the patient was flagged as “red.” The multidisciplinary team then determined that the patient should be admitted to hospital to be treated. The detailed decision-making process regarding hospitalization is presented in Figure 2. Figures 3 and 4 provide screenshots of the interfaces in which the telemedicine form is created and used, respectively.

### Composition of the Multidisciplinary Team

The multidisciplinary team consisted of multidisciplinary medical workers, including 2 physicians, 3 nurses, 1 rehabilitation physician, and 1 psychologist. The physicians gave treatment advice according to the patients’ condition updates through the eCounseling system. The nurses guided the methods of quarantine and disinfection, supervised the patient’s regular work, rest, nutrition, and diet, and urged the patients to update their conditions in the telemedicine form on a daily basis. The rehabilitation physician helped the patients develop practical rehabilitation plans, and the psychologist helped them maintain a positive mood.

### Participating Patients

The system initially recruited 188 individuals through word of mouth and physician referrals. Of these individuals, 114/188 (60.6%) showed no symptoms of COVID-19 during the 14-day quarantine period. These individuals were dismissed from the telemedicine system. The remaining 74 patients were confirmed COVID-19 cases. Among these patients, 26/74 (35%) left the system prematurely.

The multidisciplinary team followed up with every patient who dropped out; they found that all 26 patients had recovered and felt that their continued participation in the telemedicine system was unnecessary.

The observation endpoints for the patients in the telemedicine system were set as follows: patients were clinically cured (normal CT imaging reports plus a minimum of 2 rounds of negative results for SARS-CoV-2 nucleic acid detection), patients were admitted to hospital because of progression of the disease, or patients were deceased (although this did not occur in our study).

### Diagnostic Criteria, Inclusion Criteria, and Exclusion Criteria of Patients With COVID-19

The diagnostic criteria for confirmed cases were defined as the presentation of either 1 of 2 pieces of etiological evidence: testing positive for SARS-CoV-2 nucleic acid in respiratory or blood samples by reverse transcriptase–polymerase chain reaction (RT-PCR) or virus sequences detected in respiratory or blood samples sharing high homology with the known sequence of SARS-CoV-2.

The inclusion criteria of the telemedicine system were set as confirmed or suspected cases of COVID-19 and voluntary participation in medical observation. Patients who were not diagnosed with COVID-19 with certainty, pregnant or breastfeeding women, patients younger than 18 years or older than 75 years, and patients who were unable to cooperate with the data reporting were excluded from the analysis.

## Results

### Development of the Quarantine Management Assessment

We designed the quarantine management assessment scales using common symptoms presented by the patients with COVID-19 we treated at the Tongji Hospital clinic. We then gradually added new symptoms to the scales as our understanding of the disease advanced. The final version of the quarantine management assessment was designed based on these scales, which consisted of 5 primary indicators, 22 secondary indicators, and 83 tertiary indicators (Multimedia Appendix 1).

To calibrate the validity of the quarantine management assessment scales, they were submitted to 34 medical experts for evaluation. Two rounds of expert consultations were conducted. In each round, 17 experts from the emergency department, respiratory department, intensive care unit (ICU), and infectious disease department were invited to evaluate the scales. Statistical analysis of the consultation results showed that the response rates of the two rounds of expert consultations were 100% and 88.24%, respectively; the mean authoritative coefficient was 0.855. The W values for the degree of coordination of the expert opinions were 0.204 and 0.293 for the two rounds, respectively. The W values were also statistically significant (*P*=.003).

### Demographics and Baseline Characteristics on Admission

Of the 48 patients in the study, 35 (73%) were female. The median age was 37.5 years (IQR 30.00-45.00). Fever was the most common initial symptom among the 48 patients (28, 58%), followed by cough (16, 33%), lack of strength (6, 13%), muscle soreness (5, 10%), and nasal congestion (3, 6%) (Table 1). One patient had no symptoms upon their initial clinic visit. Six patients were admitted to hospital later because their conditions worsened during home quarantine; all these patients had fever. Of the hospitalized patients, 3 had coughs and 3 also showed infiltration in both lungs at the time of diagnosis. However, 2 hospitalized patients exhibited normal CT imaging at initial diagnosis (Table 2).

**Table 1:** Demographics, baseline characteristics, and clinical outcomes of the home-quarantined patients with COVID-19 (N=48).

Characteristic		All patients (N=48)	Nonhospitalized patients (n=42)	Hospitalized patients (n=6)
Age (years), median (IQR)		37.50 (30.00-45.00)	35.50 (29.50-44.25)	55.00 (37.25-70.25)
**Age (years), n (%)**				
	≤39	28 (58)	26 (62)	2 (33)
	40-49	12 (25)	11 (26)	1 (17)
	50-59	4 (8)	4 (10)	0 (0)
	60-69	1 (2)	0 (0)	1 (17)
	≥70	3 (6)	1 (2)	2 (33)
**Gender, n (%)**				
	Male	13 (27)	10 (24)	3 (50)
	Female	35 (73)	32 (76)	3 (50)
**Initial symptoms, n (%)**				
	Headache	3 (6)	3 (7)	0 (0)
	Muscle soreness	5 (10)	5 (12)	0 (0)
	Cough	16 (33)	13 (31)	3 (50)
	Diarrhea	4 (8)	4 (10)	0 (0)
	Dyspnea	1 (2)	1 (2)	0 (0)
	Sore throat	1 (2)	1 (2)	0 (0)
	Dizziness	2 (4)	2 (5)	0 (0)
	Chest tightness	4 (8)	3 (7)	1 (17)
	Fever	28 (58)	22 (52)	6 (100)
	Chest pain	1 (2)	1 (2)	0 (0)
	Nasal congestion	3 (6)	3 (7)	0 (0)
	Loss of appetite	2 (4)	2 (5)	0 (0)
	Lack of strength	6 (13)	5 (12)	1 (17)
**Number of initial symptoms, n (%)**				
	No symptoms	1 (2)	1 (2)	0 (0)
	1 symptom	27 (56)	25 (60)	2 (33)
	2 symptoms	13 (27)	10 (24)	3 (50)
	3 symptoms	5 (10)	4 (10)	1 (17)
	4 symptoms	2 (4)	2 (5)	0 (0)
**CT^a^ manifestation at onset, n (%)**				
	Normal	7 (15)	5 (12)	2 (33)
	Ground-glass opacity in one lung	13 (27)	11 (26)	2 (33)
	Ground-glass opacity in both lungs	19 (40)	19 (45)	0 (0)
	Infiltration in both lungs	9 (19)	7 (17)	2 (33)
**White blood cell count, n (%)**				
	Normal	25 (52)	22 (52)	3 (50)
	Decreased	18 (38)	17 (41)	1 (17)
	Elevated	5 (10)	3 (7)	2 (33)
**Lymphocyte count, n (%)**				
	Normal	20 (42)	20 (48)	0 (0)
	Decreased	28 (58)	22 (52)	6 (100)

^a^CT: computed tomography.

**Figure 5:**
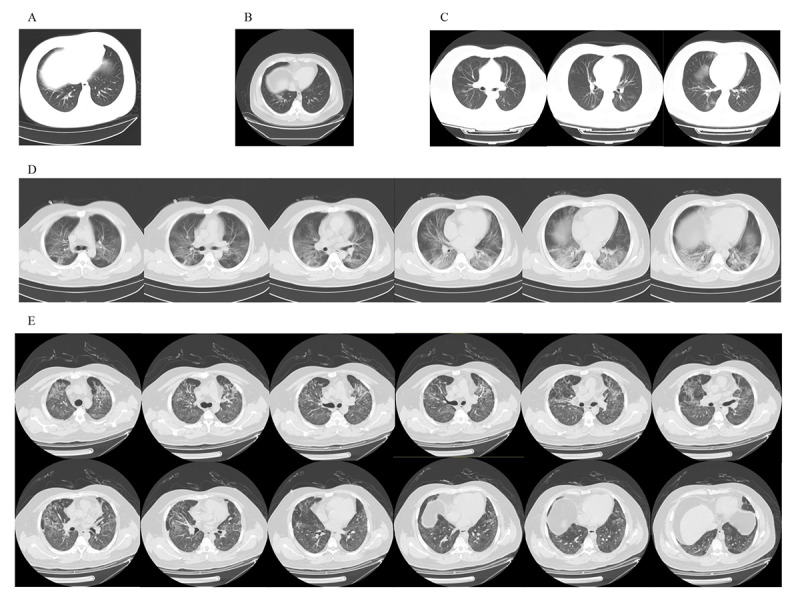
Chest computed tomography (CT) images of Patient No. 4 showing small patchy shadows on the lower right lung on day 1 (A) and day 3 (B) after symptom onset. Chest CT images showing bilateral ground-glass opacities on day 6 after symptom onset (C) and bilateral large infiltrative shadows with partial consolidation on day 10 after symptom onset (D). Chest CT images showing bilateral diffuse reticular changes and fibrous stripes on day 45 after symptom onset (E).

Of the 6 hospitalized patients, 2 patients (33%) progressed to critical condition. One patient (labeled as Patient No. 3 in Table 2) was directly admitted to the ICU. ECMO support was provided to this patient for 17 days. He was transferred to the general ward when his condition stabilized. Eventually, the patient recovered (Table 2). Another hospitalized patient (labeled as Patient No. 4 in Table 2) was found to have a persistent fever on day 5 of observation. The patient’s CT results identified ground-glass opacities in both lungs (Figure 5). Emergency hospital admission was requested for the patient. Noninvasive ventilation was provided, and the patient’s condition gradually improved. This patient was discharged from hospital after 30 days of treatment (Table 2).

**Figure 6:**
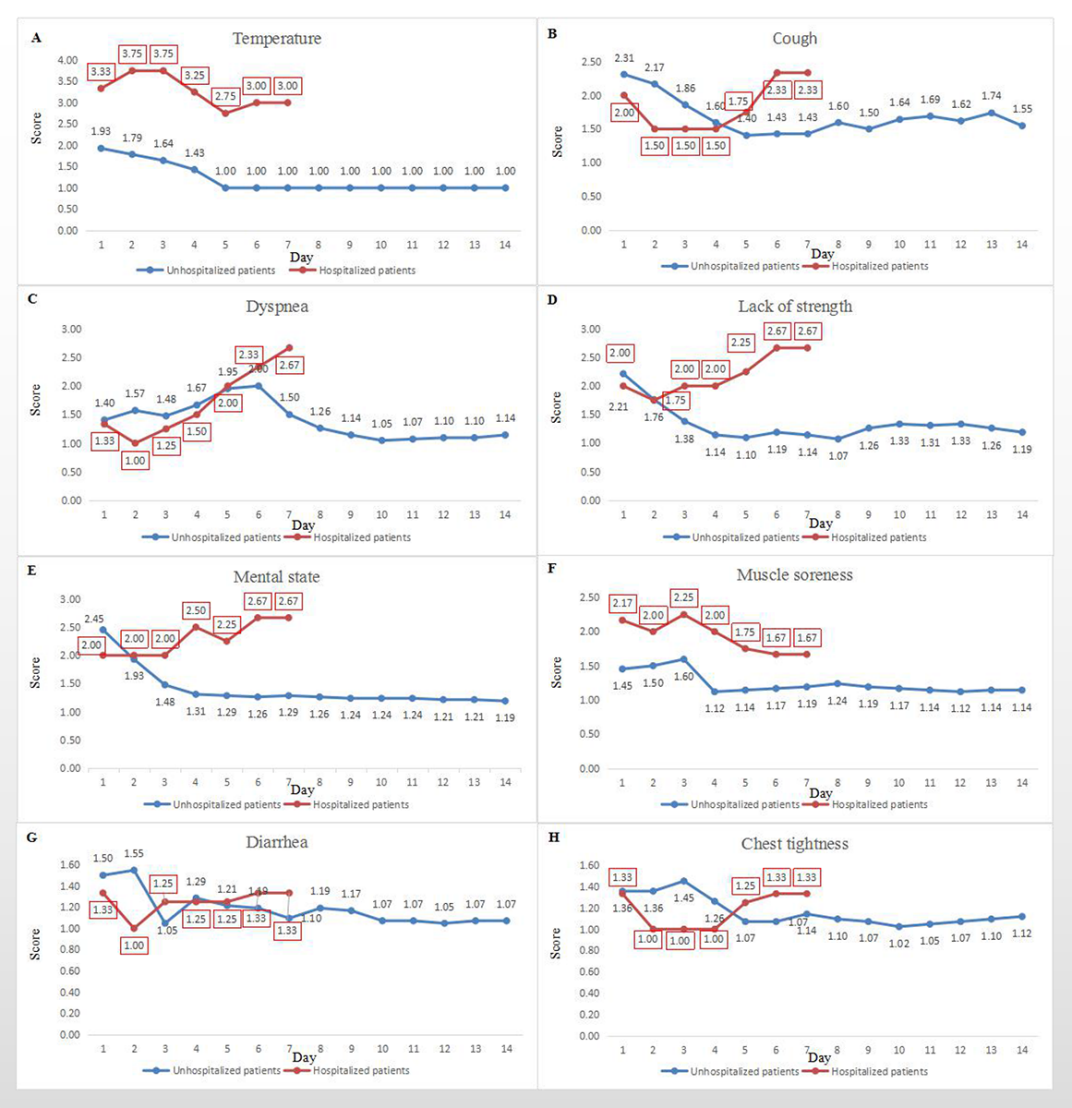
Comparison of symptom trends between non-hospitalized and hospitalized patients.

Based on the information collected through the eCounseling system, we found that there were differences in disease progression between patients with mild conditions and the patients who were hospitalized. The hospitalized patients had appreciably elevated body temperature at onset, which scored between 3 and 4 points (approximately 38-40 ºC) and persisted longer than that of nonhospitalized patients. The mean body temperature of the nonhospitalized patients became normal by day 4 or 5 (Figure 6A). In comparison, the body temperature of the hospitalized patients remained elevated on day 5 to 6 along with exacerbated cough (Figure 6B).

The results of the quarantine management assessment of symptom progression also provided direct insight into timely intervention for patients whose conditions deteriorated over time. The separation between the dyspnea curves was very distinct between nonhospitalized and hospitalized patients. For nonhospitalized patients with mild symptoms, dyspnea peaked on day 6 with a score of 2 to 3, which manifests as shortness of breath when hurrying on a level surface. However, these patients’ symptoms gradually improved over time. In contrast, hospitalized patients had sustained dyspnea that continued to worsen over time (Figure 6C).

Although the degree of lack of strength appeared to be unrelated to disease severity at onset, this symptom was never alleviated among hospitalized patients and continued to worsen over time. In comparison, among nonhospitalized patients, strength was nearly normal by day 4 (Figure 6D). In addition, we observed that the mental state of hospitalized patients gradually deteriorated over the course of the disease, showing a similar pattern to lack of strength (Figure 6E). Furthermore, while muscle soreness was alleviated on day 4 for all patients, the degree of muscle soreness in hospitalized patients continued to be higher than normal (Figure 6F). The progression of diarrhea was inconsistent for both groups (Figure 6G). This may be due to the side effects of certain antiviral drugs. We also found that 4 days after the onset of illness, the chest tightness of hospitalized patients gradually worsened (Figure 6H).

**Table 2:** Clinical characteristics of the participating patients admitted to hospital (n=6) based on the medical observation scales described in Multimedia Appendix 1.

Characteristic		Patient No. 1	Patient No. 2	Patient No. 3	Patient No. 4	Patient No. 5	Patient No. 6
Age (years)		38	67	43	35	71	70
Gender		Female	Male	Male	Male	Female	Female
**Presenting symptoms and signs at onset**							
	Fever^a^	Moderate	None	Moderate	Moderate	Low-grade	Moderate
	Cough	Frequent and slightly interferes with daily activities	Occasional	Frequent and slightly interferes with daily activities	Occasional	Frequent and slightly interferes with daily activities	Frequent and slightly interferes with daily activities
	Lack of strength	Moderate	Mild	Severe	Moderate	Mild	Severe
	Diarrhea	Moderate	Mild	Mild	Mild	Moderate	None
	Chest tightness	None	Moderate	Moderate	Moderate	Moderate	None
	Dyspnea level^b^	1	2	3	2	3	4
	Mental state	Poor	Average	Poor	Average	Average	Poor
	Muscle soreness	Occasional	None	Occasional	None	Occasional	Light
**Laboratory results at onset**							
	Procalcitonin	Normal	Normal	Normal	Normal	Normal	Normal
	White blood cell count	Normal	Normal	Elevated	Decreased	Elevated	Normal
	Lymphocyte percentage	Decreased	Decreased	Decreased	Decreased	Decreased	Decreased
CT^c^ image characteristics at onset		Few ground-glass opacities in left lung	Few ground-glass opacities in right lung	Large infiltration shadow in both lungs	Normal	Multiple infections in both lungs	Normal
Number of days since onset before admission		5	7	1	5	1	7
**Signs and symptoms at admission**							
	Fever	None	Moderate	Moderate	Low grade	Low grade	None
	Cough	None	Frequent and slightly interferes with daily activities	Frequent and slightly interferes with daily activities	Frequent and slightly interferes with daily activities	Frequent and slightly interferes with daily activities	Occasional
	Lack of strength	None	Severe	Severe	Mild	Mild	Mild
	Diarrhea	None	Mild	Mild	None	Moderate	None
	Chest tightness	None	None	Moderate	Mild	Moderate	Moderate
	Dyspnea level	1	4	3	2	3	2
	Mental state	Good	Poor	Poor	Poor	Average	Average
	Muscle soreness	Light	Light	Occasional	Light	Occasional	None
**Laboratory results at admission**							
	White blood cell count	Normal	Normal	Elevated	Normal	Elevated	Normal
	Lymphocyte percentage	Decreased	Decreased	Decreased	Decreased	Decreased	Decreased
CT image characteristics at admission		Bilateral diffused patchy shadows	Bilateral diffused patchy shadows	Bilateral infiltration shadow	Bilateral multiple patches of infiltration shadows	Bilateral multiple infections	Bilateral multiple infections
Outcome		Discharged	Discharged	Discharged	Discharged	Discharged	Discharged

^a^Fever: None (37.3 degrees Celsius and below); Low grade (37.3-38 °C); Moderate (38.1-39 ºC).

^b^Dyspnea level: 1=Not troubled by breathlessness except with strenuous exercise; 2=Troubled by shortness of breath when hurrying on a level surface or walking up a slight hill; 3=Experience breathlessness or must stop for breath when walking on a level surface at own pace; 4=Stop for breath after walking 100 meters or after a few minutes on a level surface.

^c^CT: computed tomography.

## Discussion

### Principal Findings

World Health Organization (WHO) emergency guidelines recommend considering alternative quarantine methods, including homecare and isolation, for patients with COVID-19 who have mild symptoms in cases of insufficient hospitalization conditions or medical resources [3]. However, these guidelines do not provide details on how this home quarantine should be conducted, nor do they offer instructions on what to do when patients’ conditions become severe or critical. A notable example is the British Prime Minister Boris Johnson, who was admitted to the ICU after 11 days of home quarantine [10]. More concerningly, based on initial clinic symptoms and laboratory tests, it is difficult to distinguish patients whose conditions will later become severe or critical. For example, CT results were normal for 7/48 (15%) of the patients in our study during initial diagnosis, including 2 patients who were hospitalized. Our results were consistent with the findings of Guan et al [11], who reported that CT images were normal for 17.9% of patients with mild conditions and 2.9% patients with severe conditions, respectively. Thus, it may be a common challenge to identify patients during an initial clinic screening whose conditions are prone to become severe or critical. Constant observation of home-quarantined patients by medical staff may therefore be of lifesaving importance.

The telemedicine model presented in this paper not only fills the gap in the WHO guidelines on home quarantine but also mitigates the subsequent threats of the disease caused by a lack of initial symptoms. Specifically, through the quarantine management assessment, the telemedicine system can complement the initial clinic screening and, hence, greatly increase the accuracy of diagnosis. Through the eCounseling system, the telemedicine system can detect any newly emerged symptoms; then, the multidisciplinary team can be promptly informed to make appropriate treatment decisions.

To demonstrate the merits of the telemedicine system, fever can be considered as an example. Current studies list fever as an indispensable or highly prevalent symptom during the initial phase of COVID-19 infection [12-16]. However, in the study by Guan et al [11], fever was only present in about half (48.7%) of the patients during initial diagnosis. The telemedicine system proposed in this paper helped patients report their elevated body temperatures later to medical staff, who then could monitor the changes in body temperature over time to detect any alarming patterns. We found that hospitalized patients had elevated body temperature that was sustained considerably longer than that of nonhospitalized patients. In contrast, the body temperature of nonhospitalized patients returned to normal by day 4 to 5. This difference can be detected only through continuous observation implemented by the telemedicine system or another similar system. Thus, the telemedicine system greatly aids medical staff in making correct treatment decisions without being misguided by the initial clinic diagnosis.

The telemedicine system also contributes to clinical practice by identifying the key roles of dynamic changes in four diagnostic symptoms: fever, dyspnea, lack of strength, and muscle soreness. Dyspnea peaked on day 6 for nonhospitalized patients but persisted in hospitalized patients and was exacerbated over time. Similarly, both lack of strength and muscle soreness returned to normal by day 4 for nonhospitalized patients but not for hospitalized patients. When a patient reported via the eCounseling system that she was still experiencing the symptoms listed above after day 4, the multidisciplinary team went on alert and paid closer attention to that patient.

Further, these symptoms have not been fully studied in the literature on COVID-19. Therefore, tracing dynamic changes in the abovementioned symptoms paves the way for future studies to investigate whether these time markers can serve as turning points of the disease. In comparison, we found that excessive laboratory data and physical examination were not strictly necessary to evaluate patients with mild symptoms. Instead, the evaluation could be performed through patients’ subjective initiative and active participation through self-monitoring of the disease.

Another contribution of this telemedicine system is the quarantine management assessment scales. This set of scales provides hands-on and easy-to-use self-diagnosis tools for home-quarantined patients. It also helps medical staff obtain more details about the clinical symptoms of the patients without the need for close physical contact in a clinic. We found that the quarantine management assessment worked well in identifying patients with disease progression.

From the resource management perspective, the telemedicine system enabled management of 188 individuals initially and 74 patients later by a team consisting of only 7 medical workers. None of the patients died, and none of the multidisciplinary team members were infected with COVID-19. Efficiency is important for regions whose medical workforce has been impacted by medical worker infection. Many infected medical workers were required to remain at home for at least two weeks after recovery. One merit of the telemedicine system is that medical staff who have recovered from COVID-19 and are self-quarantined can be enlisted to help. Thus, the shortage in the medical workforce can be alleviated, and other medical workers can be freed to fight COVID-19 on the front line. In conclusion, the implementation of such a system can optimize the usage of local medical resources and reduce cross-infection among medical workers and patients.

### Limitations and Future Work

One limitation of this study is that its scope was restricted by the suddenness and complexity of the COVID-19 outbreak as well as the diversity and latency of the clinical manifestations of the disease. As a result, we were unable to obtain a larger sample size to achieve a higher level of validity of the findings. It is worth mentioning that we are currently testing the telemedicine system globally. As of April 9, 2020, 1421 patients worldwide are using this system, including 804 in Italy, 250 in the United Kingdom, 181 in France, and 168 in the United States. More than 60% of the participating patients are quarantined at home. We expect to report further findings in the future.

### Conclusion

Continuous monitoring of patients with COVID-19 quarantined at home with a telemedicine system helped greatly reduce the risks of delayed hospitalization due to disease progression. Through this system, medical workers can trace changes in several key symptoms and intervene in the home quarantine in time for hospitalization. The telemedicine system proposed in this study was proven to be effectual and efficient. Implementation of this system will optimize the usage of local medical resources and reduce cross-infection among medical workers and patients.

## Appendix

Multimedia Appendix 1 Quarantine management assessment for home-quarantined patients with COVID-19.

## Abbreviations

COVID-19: coronavirus disease
CT: computed tomography
ECMO: extracorporeal membrane oxygenation
eCounseling: electronic counseling
ICU: intensive care unit
RT-PCR: reverse transcriptase–polymerase chain reaction
SARS-CoV-2: severe acute respiratory syndrome coronavirus 2
WHO: World Health Organization
