# Supplementary material for: Monitoring and Management of Home-Quarantined Patients With COVID-19 Using a WeChat-Based Telemedicine System: Retrospective Cohort Study
Source: J Med Internet Res. 2020 Jul 2;22(7):e19514. doi: 10.2196/19514 (PMC7333794; doi:10.2196/19514)
Supplement: Multimedia Appendix 1 [file jmir_v22i7e19514_app1.doc]

| **Quarantine Management Assessment for Home Quarantined COVID-19 Patients** | | | | | | | | | | | | | | | | | |
| --- | --- | --- | --- | --- | --- | --- | --- | --- | --- | --- | --- | --- | --- | --- | --- | --- | --- |
| **General information** | | | | | | | | | | | | | | | | | |
| Name |  | Sex |  | Age | | |  | | Pregnant or not | |  | | Status | |  | Comorbidities |  |
| Contact history | | | |  | | | | | Medicine | | | |  | | Prednisone | |  |
| Symptom |  | | | Date of onset | | |  | | CT scan | | | |  | | | | |
| C-reactive protein |  | Procalcitonin |  | Nucleic acid test | | |  | | White blood cell（10^9/L） | | |  | Eosnophils（10^9/L） | |  | Lymphocyte  （10^9/L） |  |
| **Medical observation scale for patients quarantined at home** | | | | | | | | | | | | | | | | | |
| **Please refer to the following instructions for item A to H and fill in the forms day by day.** | | | | | | | | | | | | | | | | | |
| A.Fever: 1 = None (37·3℃ and below); 2 = Low grade fever (37·3～38℃); 3 = Moderate fever (38·1～39℃); 4 = High fever (39·1～40℃); 5 = Hyperpyrexia (40℃ and higher) | | | | | | | | | | | | | | | | | |
| B.Mental state: 1 = Good; 2 = Average; 3 = Poor | | | | | | | | | | | | | | | | | |
| C.Muscle soreness: 1 = Complete absence of soreness; 2 = Light pain felt only when touched; 3 = Occasional soreness; 4 = Sustained soreness | | | | | | | | | | | | | | | | | |
| D.Cough: 1 = None; 2 = Occasional; 3 = Frequent and slightly interferes with daily activities; 4 = Frequent and seriously interferes with daily activities | | | | | | | | | | | | | | | | | |
| E.Dyspnea: 1 = Not troubled by breathlessness except with strenuous exercise; 2 = Troubled by shortness of breath when hurrying on a level surface or walking up a slight hill; 3 = Experience breathlessness or has to stop for breath when walking on level surface at own pace; 4 = Stop for breath after walking 100 meters or after a few minutes on a level surface; 5 = Too breathless to leave the house | | | | | | | | | | | | | | | | | |
| F.Lack of strength: 1 = No lack of strength; 2 = Mild: Slightly lacked strength, able to do physical work, improves after rest but does not recover to normal; 3 = Moderate: Lacked strength, feels weak, able to persist in daily activities and work but light physical work is very tiring and does not recover to normal after long periods of rest; 4 = Severe: Extremely lacked strength, unable to conduct normal activities, feels tired at rest, cannot talk | | | | | | | | | | | | | | | | | |
| G.Diarrhea: 1 = No diarrhea; 2 = Mild diarrhea: Loose stool for no more than 3 times; 3 = Moderate diarrhea: 4-6 times; 4 = Severe diarrhea: More than 6 times | | | | | | | | | | | | | | | | | |
| H.Chest tightness: 1 = None; 2 = Mild; 3 = Moderate; 4 = Severe | | | | | | | | | | | | | | | | | |
| Date | Time | A.  Fever | B.  Mental state | | C.  Muscle soreness | D.  Cough | | E.  Dyspnea | | F.  Lack of strength | | | | G.  Diarrhea | H.  Chest tightness | | Test results |
|  |  |  |  | |  |  | |  | |  | | | |  |  | |  |
|  |  |  |  | |  |  | |  | |  | | | |  |  | |  |
|  |  |  |  | |  |  | |  | |  | | | |  |  | |  |
|  |  |  |  | |  |  | |  | |  | | | |  |  | |  |
|  |  |  |  | |  |  | |  | |  | | | |  |  | |  |
|  |  |  |  | |  |  | |  | |  | | | |  |  | |  |
